# Evaluation of two gradient diffusion tests to determine susceptibility to aztreonam and ceftazidime–avibactam in combination

**DOI:** 10.1128/aac.01736-24

**Published:** 2025-02-07

**Authors:** Ayesha Khan, Carmila Manuel, Richard Maynard, Romney M. Humphries

**Affiliations:** 1 Pathology, Microbiology and Immunology, Vanderbilt University Medical Center 204907 , Nashville, Tennessee, USA; Johns Hopkins University School of Medicine, Baltimore, Maryland, USA

**Keywords:** antibiotic resistance, susceptibility testing, multidrug resistance, metalloenzymes, antimicrobial combinations, antimicrobial agents, diagnostics, *Stenotrophomonas*, Enterobacteriaceae, *Pseudomonas aeruginosa*

## Abstract

The combination of aztreonam and ceftazidime–avibactam (ATM–CZA) is a last resort regimen against recalcitrant infections caused by metallo-β-lactamase (MBL)-producing organisms. Susceptibility testing is warranted due to emerging resistance to the combination, but there are no widely implemented methods for use in clinical laboratories. Here, we used a cohort of 100 Enterobacterales*, Pseudomonas aeruginosa*, and *Stenotrophomonas maltophilia* strains, including 68 MBL producers, to evaluate the performance of two ETEST strip-based synergy testing methods: the side-by-side (SS) method with an ATM ETEST placed next to a CZA ETEST (10 mm apart) and the strip cross (SX) method with a CZA ETEST placed perpendicularly on top of the ATM ETEST (at the 8 µg/mL mark). By reference broth microdilution (BMD), 89.1% (41/46) of the Enterobacterales, 15% (3/20) of the *P. aeruginosa*, and 97.1% (33/34) of the *S. maltophilia* isolates tested susceptible to the ATM–CZA combination. The SS method yielded 72% categorical agreement with BMD and 28 major errors (ME, 36.4%). Initial testing with the SX method yielded three ME , of which one was resolved upon repeat testing, yielding a final categorical agreement of 98% with BMD with two ME (2.6%). The SX method also yielded 100% reproducibility across three brands of Mueller Hinton agar (BD, Hardy, Remel). Our study demonstrates that the SX method is accurate, precise, and feasible for clinical laboratories to perform ATM–CZA susceptibility testing to guide use of the combination for treatment of multidrug-resistant gram-negative pathogens.

## INTRODUCTION

Antimicrobial resistance is a major global public health threat. Carbapenemase-producing organisms (CPO) are of particular concern due to the sparsity of treatment options for these organisms (1). In the United States, carbapenem resistance among the Enterobacterales is often due to the production of *Klebsiella pneumoniae* carbapenemase (KPC), a serine carbapenemase. Treatment outcomes for patients with infections caused by KPC-producing Enterobacterales were dramatically improved with the introduction of beta-lactam combinations (BLCs) with activity against serine carbapenemases, including ceftazidime–avibactam (CZA), meropenem–vaborbactam, and imipenem–relebactam. The novel BLCs are now the standard of care for the treatment of KPC-producing organisms, with multiple studies showing 50–60% decrease in mortality following infection over best alternative therapies, which often include colistin in combination with other agents (2–9). Despite these advances, CPO remain a threat to patient and public health. The incidence of metallo-β-lactamases (MBLs), which hydrolyze all beta-lactams, except aztreonam (ATM), and are not inhibited by the novel BLCs, has increased markedly in the US between 2019 and 2021 (10, 11). Furthermore, MBLs are the most common carbapenemase in Enterobacterales isolated from patients in Eastern Europe and Southeast Asia. Invasive infections caused by MBL producers demonstrate treatment failure rates and mortalities in the range of those observed for KPC producers prior to the introduction of the novel BLCs (12). Infections caused by *Stenotrophomonas maltophilia*, an intrinsically MBL-expressing organism, *Stenotrophomonas maltophilia* are similarly associated with high rates of treatment failure. The incidence of *S. maltophilia* infections is increasing in patients with hematological malignancies (13).

In the absence of other options, combination therapy with ATM and CZA or cefiderocol monotherapy is recommended by the Infectious Diseases Society of America as first-line for the treatment of MBL-producing bacteria, including Enterobacterales*, Pseudomonas aeruginosa*, and *S. maltophilia* (14, 15). In this combination, avibactam inhibits serine carbapenemase (e.g., KPC, OXA-48) or extended spectrum beta-lactamases that are often co-produced by MBL-producing organisms, shielding and preserving the antimicrobial activity of ATM. Phase 3 clinical trials demonstrate the ATM–avibactam combination is safe and effective in treating MBL-producing organisms but is not yet available for clinical use in the US. Furthermore, this combination is unlikely to be available in countries with the highest incidence of MBLs, such as South Korea and Japan, where CZA has only been licensed for human use in the past year (16, 17). As such, the ATM–CZA combination is likely to be used globally for some time.

Emerging resistance to ATM–avibactam has been identified (18, 19). In MBL-producing *Escherichia coli*, ATM–avibactam resistance was shown to be caused by mutations to penicillin-binding protein 3, combined with increased production of β-lactamases that hydrolyze ATM, including CMY, CTX-M, and KPC variants (18, 20). In *K. pneumoniae*, resistance was shown to be associated with downregulation or deletion of OmpF, OmpC, and/or OmpK37 porins, alongside the overexpression of ATM-hydrolyzing β-lactamases, including KPC-2, DHA-1, or CMY (20, 21). These mechanisms similarly abolish the activity of the triple combination, CZA–ATM. Thus, susceptibility testing of ATM–avibactam and ATM–CZA is critical to guide appropriate clinical use of these combinations.

Most clinical laboratories identify MBL-producing isolates using molecular, lateral flow, or functional assays. However, reliable and practical testing methods to guide clinical use of the ATM–CZA combination are not widely available, meaning this combination is often used empirically. A pragmatic approach is needed for clinical laboratories to test MBL-producing isolates for susceptibility to the ATM–CZA combination. To this end, a 2021 proof-of-concept study evaluated two Kirby Bauer disk-based methods (disk stacking, broth disk elution) and two gradient-strip based methods (strip stacking, strip crossing) against a representative cohort of multidrug-resistant CRE and CRPA isolates (22). The broth disk elution and strip crossing methods yielded the best performance with 100% sensitivity, specificity, and reproducibility relative to reference broth microdilution. In 2023, Clinical and Laboratory Standards Institute volunteers conducted a multicenter study aimed at evaluating a modification of the broth disk elution method (23). While the performance was generally good, the study found a significant inter-assay variability associated with the brand of disk or the cation-adjusted Mueller Hinton broth (24). Furthermore, this method requires roughly 30 min of hands-on time and access to supplies that may not be present in all clinical laboratories, such as the cation-adjusted Mueller Hinton broth. Laboratories routinely use gradient diffusion strips to perform antimicrobial susceptibility testing, including for novel agents, such as CZA, which may not be on commercial automated test platforms. Thus, we evaluated the performance of two gradient strip methods (strip cross and side-by-side strips), testing the *in vitro* synergy of ATM and CZA relative to reference broth microdilution (BMD) MICs with a cohort of 100 Enterobacterales*, P. aeruginosa*, and *S. maltophilia* isolates.

## MATERIALS AND METHODS

### Study design

A collection of 100 isolates were used to evaluate two gradient diffusion synergy testing methods, which are described in more detail below. Both ATM–CZA gradient diffusion synergy methods were set up in parallel with gradient diffusion for CZA and ATM alone and reference BMD using the same suspension of bacteria, which was adjusted to be equivalent to a 0.5 McFarland standard. Both methods were initially evaluated against 100 isolates. The best performing method was then selected for repeat testing of isolates that generated discordant results between the test method and BMD to rule out random testing errors. Errors that persisted on repeat testing were included in the final analysis, whereas those that resolved were removed from analysis. The breakpoints used in data analysis are listed in Table 1.

**TABLE 1:** CLSI clinical breakpoints for aztreonam and ceftazidime–avibactam and proposed breakpoints for aztreonam–avibactam^*a*^ (19)

Organism	Breakpoint (μg/mL) for:					
	Aztreonam			Ceftazidime–avibactam		Aztreonam–avibactam^*b*^
	S	I	R	S	R	S
Enterobacterales	≤4	8	≥16	≤8/4	≥16/4	≤8/4
*P. aeruginosa*	≤8	16	≥32	≤8/4	≥16/4	≤8/4
*S. maltophilia*	-^*c*^	-	-	-	-	≤8/4

^*a*^ S, susceptible; I, intermediate; R, resistant. CLSI, Clinical and Laboratory Standards Institute.

^*b*^ The proposed aztreonam–avibactam breakpoints were used to interpret ATM–CZA MIC values. MIC results >8/4 µg/mL were categorized as resistant for data analysis.

^*c*^ -, no values set.

Sixteen representative isolates were selected to evaluate reproducibility of the best-performing gradient diffusion synergy method. Each isolate was tested across three commercial MHA brands (Remel, Lenexa, Kansas; Hardy, Santa Maria, CA; BD, Franklin Lakes, NJ) inoculated with the same 0.5 McFarland suspension.

### Bacterial isolates

A total of 100 isolates were tested: two Enterobacterales American-type Culture Collection (ATCC) quality control (QC) strains, 44 isolates of Enterobacterales from the Centers for Disease Control and Prevention and US Food and Drug Administration Antibiotic Resistance (AR) Isolate Bank*,* 34 clinical isolates of *S. maltophilia* recovered from blood cultures at Vanderbilt University Medical Center, and 20 isolates of *P. aeruginosa* from the AR Bank. Of these, 68 isolates harbored MBLs, and 32 isolates did not. Nine *P. aeruginosa* isolates harbored Guiana extended-spectrum (GES) beta-lactamase variants, which have been reported to produce an MBL-like susceptibility phenotype (25). *E. coli* ATCC 25922 (susceptible to all agents), *K. pneumoniae* BAA-2146 (New Delhi metallo-β-lactamase [NDM] producer, non-susceptible to ATM and CZA individually but susceptible to the ATM–CZA combination), and *E. coli* AR348 obtained from the AR Bank (non-susceptible to ATM, CZA, and ATM–CZA) were included as QC strains to assess expected ATM–CZA susceptibility phenotype in the reference BMD and both gradient diffusion methods. QC strains were set up with each day of testing.

### Reference broth microdilution method

Reference BMD was performed with the cation-adjusted Mueller Hinton broth (BD, Sparks, MD) according to M07 guidelines with in-housed prepared panels (26). The Enterobacterales and *P. aeruginosa* isolates from the Centers for Disease Control and Prevention (CDC) AR Bank were tested on in-house BMD panels with 0.6 to 64 µg/mL ATM (Sigma, St. Louis, MO) and ATM–CZA (ceftazidime from Sigma and avibactam from MedChemExpress, Princeton, NJ). Reference BMD with CZA was performed at the CDC for the AR Bank isolates. In the ATM–CZA combination, ceftazidime and avibactam were held constant at 8 and 4 µg/mL, respectively, whereas ATM was serially diluted from 64 to 0.5 µg/mL. The *S. maltophilia* clinical isolates were tested on in-house prepared BMD panels with ATM, CZA (Sigma, St. Louis, MO), and ATM–CZA. Panels were stored at −80°C prior to use. BMD panels were incubated at 35 ± 2°C in ambient air for 16 to 20 h for *E. coli* and *P. aeruginosa* and 24 h for *S. maltophilia*.

### Gradient diffusion synergy methods

**Fig 1:**
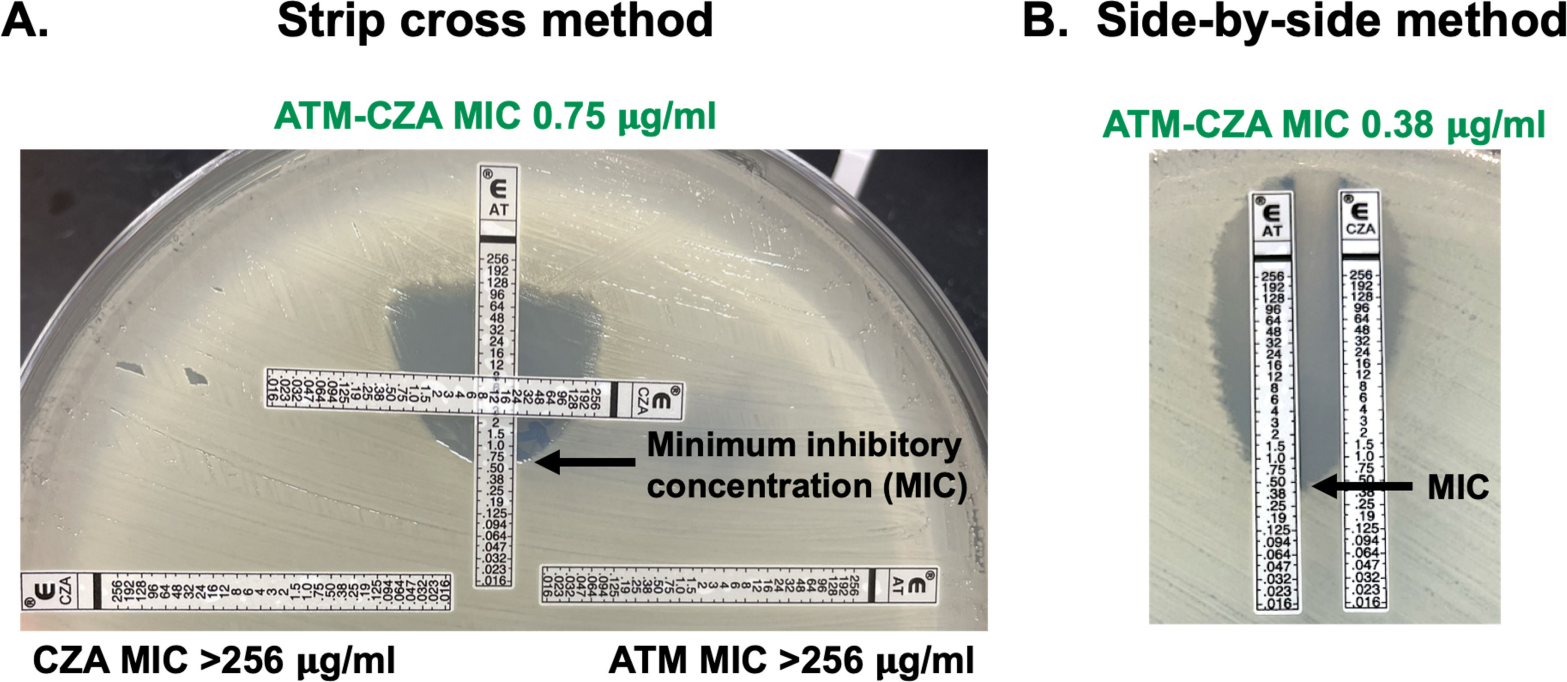
Gradient strip-based synergy testing methods displayed with a representative *E. coli* strain harboring NDM that tested susceptible to the ATM–CZA combination by broth microdilution. (**A**) Strip cross method with the CZA strip overlaid perpendicular to the AT strip. (**B**) Side-by-side strip method with AT and CZA strips placed side-by-side with 10 mm in between strips.

The two synergy testing methods evaluated in this study are displayed in Fig. 1. Gradient diffusion strips (ETEST, bioMerieux, Durham, NC) were used off-label. For both methods, Mueller Hinton agar (MHA, Remel, Lenexa, KS) was inoculated with a sterile cotton swab moistened with the same 0.5 McFarland suspension used for BMD. Gradient diffusion strips were subsequently added with sterile forceps. The protocol for the cross strip (“SX”) method (Fig. 1A) was originally optimized in a previously published proof-of-concept study with 16 representative Enterobacterales and *P. aeruginosa* isolates by A. Khan et al., which demonstrated 100% sensitivity, specificity, and reproducibility (22). Due to this precedence, we chose to validate the same method against our larger cohort of 100 Enterobacterales, *P. aeruginosa*, and *S. maltophilia* isolates. The ATM strip was placed on the plate first, followed by the CZA strip, which was crossed perpendicular to the ATM strip at the 8 µg/mL mark that is the proposed ATM–avibactam susceptible breakpoint for all organisms tested (Table 1). Plates were incubated in the same conditions and for the same duration as the reference BMD. The ATM–CZA MIC was read as the value where the growth inhibition ellipse intersects the ATM strip.

For the side-by-side (“SS”) method (Fig. 1B), ATM and CZA strips were placed next to each other in parallel with 10 mm of space in-between. This optimal distance was determined in a series of titration experiments that assessed 7, 10, 15, 20, 25, and 30 mm distances between the gradient strips with the three QC strains listed above (data not shown). The strips could be placed 10 mm apart with relative technical ease, and the distance yielded the most accurate reproducible results. The ATM–CZA MIC was read as the value where the inner ellipse formed in-between the two strips intersected with the ATM strip. The zones of inhibition were generally asymmetrical on either side of the strips, and the MIC value on the inner ellipse was used.

### Data analysis

BMD MIC values were used as a reference to evaluate the performance of the SS and SX synergy testing methods. MIC results were interpreted according to Clinical and Laboratory Standards Institute (CLSI) M100 standards (Table 1). The proposed ATM–avibactam breakpoints for Enterobacterales and *P. aeruginosa* were used to interpret ATM–CZA MIC results (27–29). *P. aeruginosa* breakpoints were utilized to analyze *S. maltophilia* due to the absence of clinical breakpoints. Categorical agreement with BMD was determined for each method. Very major errors (VMEs) were defined as isolates that tested resistant by reference BMD but susceptible by the gradient diffusion method under evaluation. Major errors (MEs) were defined as isolates that tested susceptible by reference BMD but resistant by the test method (17). Essential agreement was evaluated for the reproducibility study and calculated by determining if the gradient diffusion-obtained MIC for ATM–CZA was within one doubling dilution of the mode of the three replicate results.

## RESULTS

### Characterization of isolates by reference broth microdilution

**Fig 2:**
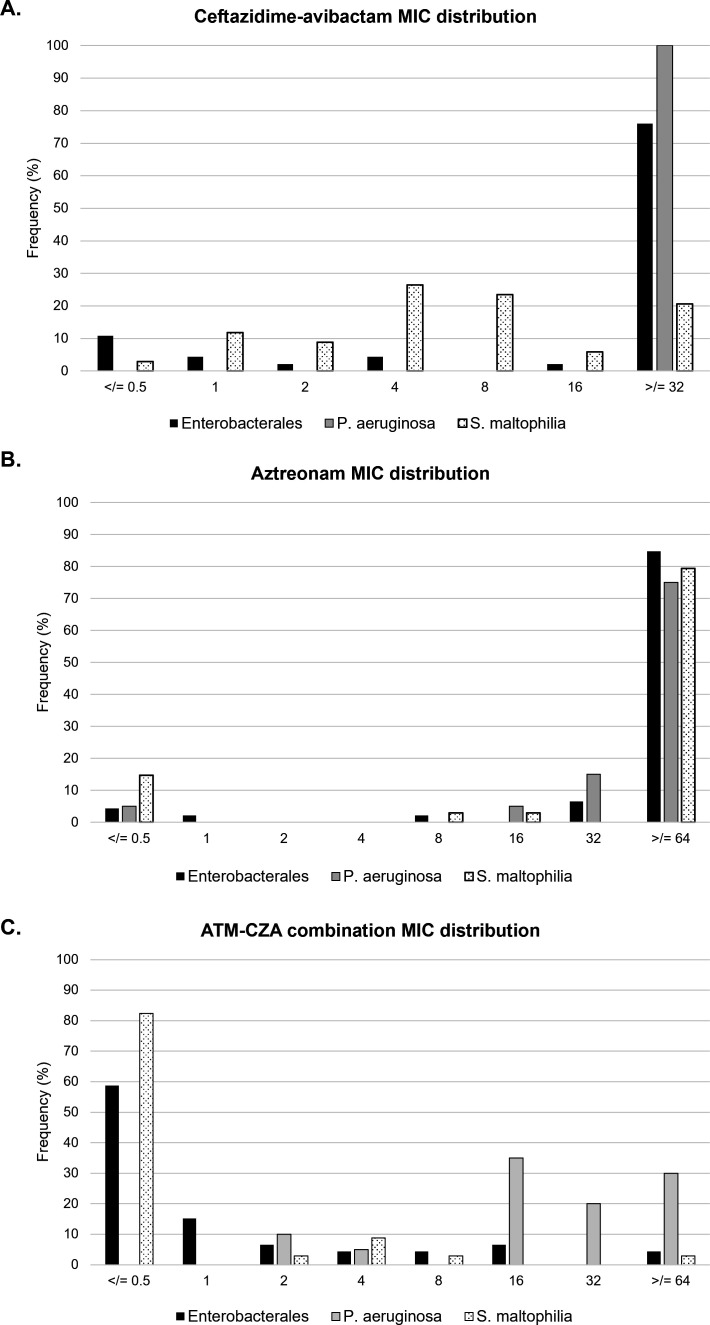
Comparing the distributions of BMD MIC values for ATM, CZA, and ATM–CZA across the Enterobacterales (*n* = 46), *P. aeruginosa* (*n* = 20), and *S. maltophilia* (*n* = 34) included in this study.

Fig. 2 shows a distribution of the ATM, CZA, and ATM–CZA MIC values against the Enterobacterales*, P. aeruginosa*, and *S. maltophilia* strains in this study. Out of the 100 isolates tested, 61 were non-susceptible to both ATM (intermediate or resistant) and CZA (resistant) (Table 2). Of these, 39 isolates (63.9%) were susceptible to the ATM + CZA combination (MIC ≤ 8 µg/mL), including 32 MBL producers, three GES producers, three KPC producers, and one AR Bank isolate that did not have any identified β-lactamases. The remaining 22 isolates (36.1%) were non-susceptible to the ATM–CZA combination (MIC > 8 µg/mL). Table 3 includes a breakdown of all isolates that were non-susceptible to both ATM and CZA individually (i.e., the isolates for which the ATM–CZA combination may have utility) parsed by β-lactamase presence.

**TABLE 2:** Isolates evaluated in this study with broth microdilution results interpreted using breakpoints listed in Table 1^*a*^

Organisms		Aztreonam (ATM)^*b*^			Ceftazidime–avibactam (CZA)^*c*^		ATM–CZA combination^*b*^	
		S	I	R	S	R	S	R
Enterobacterales	*E. coli*	1	1	9	2	9	6	5
	*Klebsiella pneumoniae*	1	0	20	3	18	21	0
	*Klebsiella aerogenes*	1		2	2	1	3	0
	*Enterobacter*	0	0	6	1	5	6	0
	*Citrobacter*	2	0	0	1	1	2	0
	*Providencia*	1	0	0	0	1	1	0
	*Morganella*	0	0	1	0	1	1	0
	*Salmonella*	0	0	1	0	1	1	0
	Total	6	1	39	9	37	41	5
*P. aeruginosa*		1	1	18	0	20	3	17
*S. maltophilia^d^*		2	1	31	25	9	33	1
All isolates		9	3	88	34	66	77	23

^*a*^ S, susceptible; I, intermediate; R, resistant.

^*b*^ MIC value determined by broth microdilution (BMD) performed in-house.

^*c*^ All isolates based on the reference MIC value provided by CDC AR Bank isolates, except *S. maltophilia* for which MICs were obtained by reference BMD performed in-house.

^*d*^ Due to the absence of CLSI M100 breakpoints for *S. maltophilia*, *P. aeruginosa* breakpoints were applied for analysis.

**TABLE 3:** Distribution of BMD results for the ATM–CZA combination for the 61 study isolates that tested non-susceptible to both ATM and CZA individually, as categorized by their β-lactamase genotype

Organisms	β-Lactamase^*a*^	Total^*b*^	ATM–CZA susceptibility by BMD	
			S (%)	R (%)
Enterobacterales	MBL	28	26 (92.9)	2 (7.1)
	Non-MBL	7	4 (57.1)	3 (42.9)
	Total	35	30 (85.7)	5 (14.3)
*P. aeruginosa*	MBL	5	0	5 (100)
	GES	8	3 (37.5)	5 (62.5)
	Non-MBL, non-GES	6	0	6 (100)
	Total	19	3 (15.8)	16 (84.2)
*S. maltophilia*	L1	7	6 (85.7)	1 (14.3)
All organisms		61	39 (64)	22 (36)

^*a*^ MBL, metallo-beta-lactamase; GES, Guiana extended-spectrum beta-lactamase.

^*b*^ Non-susceptible to ATM alone and CZA alone.

The MIC_50_/MIC_90_ values for ATM, CZA, and ATM–CZA against Enterobacterales were ≥64/≥64, ≥16/≥16, and ≤0.5/8 µg/mL, respectively. Of the 35 Enterobacterales that were non-susceptible to both ATM and CZA individually, 30 isolates (85.7%) were susceptible to ATM–CZA (26 MBL producers, three KPC producers, one without identified β-lactamases), and five isolates (14.3%) were non-susceptible to the combination (two MBL producers, two CMY producers, one without identified β-lactamases). The MIC_50_/MIC_90_ values for ATM, CZA, and ATM–CZA against *P. aeruginosa* were ≥64/≥64, ≥16/≥16, and 16/>64 µg/mL, respectively. Of the 19 *P*. *aeruginosa* strains that were non-susceptible to both ATM and CZA individually, three GES-producing isolates (15.8%) were susceptible to ATM–CZA, while the remaining 16 isolates (84.2%) were not susceptible to the combination (five MBL producers, five GES producers, five OXA producers, one without identified β-lactamases). Overall, 89.1% (41/46) of the Enterobacterales strains and 15% (3/20) of the *P. aeruginosa* strains in this study tested susceptible to the ATM–CZA combination. *P. aeruginosa* breakpoints were used to analyze *S. maltophilia* results due to the absence of organism-specific breakpoints. The MIC_50_/MIC_90_ values for ATM, CZA, and ATM–CZA against these 34 strains were ≥64/≥64, 4/≥64, and ≤0.5/4 µg/mL, respectively. Thus, independent of the interpretation, a significant reduction in ATM MIC was observed in the presence of CZA. Overall, 97.1% (33/34) of the *S. maltophilia* strains tested susceptible to the ATM–CZA combination.

### Performance of the side-by-side gradient strip method relative to broth microdilution

We evaluated performance of the SS method relative to reference BMD. The categorical agreement was 72%, with 28 major errors (36.4%) and no very major errors (Table 4). ME were observed for 12 Enterobacterales*,* three *P*. *aeruginosa*, and 13 *S*. *maltophilia*. The essential agreement was 31.4% (*n* = 11) for the 35 isolates with evaluable on-scale MICs by BMD out of the 100 isolates tested. The SS method yielded MIC values that were the same for one isolate, one doubling dilution above for 10 isolates, two dilutions above for five isolates, three dilutions above for two isolates, and four or more dilutions above for 17 isolates. The 24 isolates out of essential agreement (i.e., MIC by SS method is ≥2 log_2_ dilution higher than BMD) included 12 Enterobacterales, two *S*. *maltophilia*, and 10 *P*. *aeruginosa* strains. Of these 24 isolates, 12 displayed a categorical agreement between MICs, including five Enterobacterales and seven *P*. *aeruginosa* isolates. The performance of the SS method was unacceptable due to the exceedingly high ME rate; thus, repeat testing was not performed.

**TABLE 4:** Summary of the performance of the side-by-side and strip cross methods relative to broth microdilution^*a*^

Organism	#	S	R	Side-by-side method			Strip cross method (following resolution)		
				CA (%)	VME	ME (%)	CA (%)	VME	ME (%)
Enterobacterales	46	41	5	34 (73.9)	0	12 (29.3)	44 (95.7)	0	1 (2.4)
*P. aeruginosa*	20	3	17	19 (95)	0	1 (33.3)	19 (95)	0	1 (33.3)
*S. maltophilia*	34	33	1	21 (61.8)	0	13 (39.4)	34 (100)	0	0
All isolates	100	77	23	74 (74)	0	26 (33.8)	97 (97)	0	2 (2.6)

^*a*^ #, number of isolates; S, susceptible; R, resistant; CA, categorical agreement; VME, very major error; ME, major error.

### Performance of the strip cross gradient strip method relative to broth microdilution

Initial testing resulted in 97% categorical agreement, with three ME (3.9%) and no VME, between the SX method and reference BMD. One ME was from a GES-producing *P. aeruginosa* isolate (AR768) that yielded an ATM–CZA MIC of 4 µg/mL by BMD and an MIC ≥ 256 µg/mL by the SX method. This error persisted upon repeat testing. This isolate also yielded an ME by the SS method with an MIC ≥ 256 µg/mL. The other two ME were *K. pneumoniae* isolates harboring KPC-3. One of the two errors (AR453) was resolved upon repeat testing. AR453 had a BMD MIC of 2 µg/mL, with the SX method yielding an MIC ≥ 256 µg/mL initially and an MIC of 4 µg/mL on repeat testing. AR347 had a BMD MIC of 8 µg/mL, and the SX method initially yielded an MIC of ≥256 µg/mL, which was confirmed on repeat testing. The final analysis of the SX method after repeat testing resulted in 98% overall categorical agreement, with two ME (2.6%) relative to BMD and 62.9% (22/35) evaluable essential agreement (Table 4). The 13 isolates out of essential agreement included four Enterobacterales and nine *P*. *aeruginosa* strains. Of these 13 isolates, 11 displayed MICs in categorical agreement.

### Reproducibility testing

The SX method was evaluated for reproducibility. Sixteen isolates were tested by the SX method across three brands MHA (Hardy, BD BBL, Remel) using the same inoculum. We included 11 isolates that were susceptible and five isolates that were resistant to the ATM–CZA combination. The reproducibility was calculated as essential agreement. The overall reproducibility by the SX method was 100% (Table 5).

**TABLE 5:** Reproducibility testing results across three brands of commercial Mueller Hinton agar—Hardy Diagnostics, Becton–Dickinson, and Thermo Fisher Scientific (Remel)

Isolate	Susceptibility phenotype	Strip cross MIC (μg/mL)		
		Hardy	BD	Remel
*E. cloacae* AR38	ATM resistant, CZA resistant ATM–CZA combination susceptible	1	1	1
*K. pneumoniae* AR40		1	1	1
*E. coli* AR48		4	4	4
*M. morganii* AR57		0.25	0.25	0.25
*K. pneumoniae* AR68		2	2	2
*K. aerogenes* AR161		0.25	0.25	0.25
*K. pneumoniae* AR453		2	2	2
*S. maltophilia* 160		4	4	4
*E. coli* AR1055	ATM resistant, CZA resistant ATM–CZA combination resistant	≥256	≥256	≥256
*P. aeruginosa* AR235		≥256	≥256	≥256
*E. coli* AR348		≥256	≥256	≥256
*P. aeruginosa* AR1112		128	64	64
*E. coli* AR434		≥256	≥256	≥256
*S. maltophilia* 159	ATM resistant, CZA susceptible ATM–CZA combination susceptible	0.5	0.5	0.5
*E. cloacae* AR02		1	1	1
*K. pneumoniae* AR03		2	2	1

## DISCUSSION

The combination of aztreonam and ceftazidime–avibactam (ATM–CZA) has been shown to be effective as a last-resort treatment regimen against MBL-producing organisms. However, there are no validated and widely implemented testing methods for clinical laboratories to determine susceptibility to this combination. In our study, we evaluated the performance of two ETEST strip-based synergy testing methods: the side-by-side (SS) method with an ATM ETEST placed next to a CZA ETEST (10 mm apart) and the strip cross (SX) method with a CZA ETEST placed perpendicularly on top of the ATM ETEST (at the 4–8 μg/mL mark). This study was conducted through our laboratory’s need to determine CZA–ATM susceptibility for MBL-producing isolates, including *S. maltophilia*. The SS method was conceived through discussions with collaborators in low- and middle-income countries, for whom using the three ETEST strips required at minimum in the SX method (one for the individual ATM MIC and two for the ATM–CZA combination) was cost prohibitive. In concept, by laying an ATM strip parallel to a CZA strip , one could measure the individual agent’s MIC on the outer ellipse and the ATM–CZA combination MIC on the inner ellipse of growth inhibition with two total ETEST strips. Though our laboratory found this “SS” method easy to perform, and early data showed promise, a more robust evaluation conducted herein did not show acceptable performance, yielding excessively high ME rates. Additionally, evaluable essential agreement was low (31.4%, 11/35), and the SS method consistently over-called MICs compared to BMD.

In contrast, the SX method was accurate and reproducible across a variety of MHA media. In this study, we found overall categorical agreement of 97% with only two (2.6%) ME. The SX method is feasible for implementation in clinical laboratories because it utilizes routine susceptibility testing reagents with a minimal hands-on time, familiar result reading process, and MIC-based interpretation of results. Unlike other described methods, which require pre-knowledge of the CZA and ATM MICs (the point at which the strips are crossed), the SX evaluated herein was done by laying the strips across each other at a single point. While precisely placing the strips in this orientation was somewhat challenging for some of our technologists, we developed a template, whereby guidelines were visible through the agar to accurately place the strips.

Laboratories considering implementing the SX method for ATM–CZA susceptibility testing should devise a testing and reporting algorithm to ensure appropriate assay utilization. Our recommendations are summarized in Table 6. ATM–CZA susceptibility testing has the most clinical utility for carbapenem-resistant Enterobacterales that test resistant to both ATM and CZA individually. Importantly, this is not only in the context of MBLs for Enterobacterales, as resistance to these agents may occur due to ESBL and PBP3 mutants. As neither ATM nor CZA harbors individual activity against isolates of *S. maltophilia*, testing of CZA–ATM should be considered when this combination is being considered for therapy. Testing is also warranted for any Enterobacterales isolates with confirmed molecular or phenotypic detection of an MBL enzyme or prospectively for *S. maltophilia* isolates that warrant AST. For example, the SX method can be set up alongside routine susceptibility testing for a bloodstream Enterobacterales isolate where direct genotypic testing from a positive blood culture detected an MBL enzyme or for an Enterobacterales isolate identified as an MBL producer with a biochemical assay (e.g., EDTA carbapenem inactivation method) or a lateral flow immunoassay (e.g., CARBA-5, Hardy, Santa Maria, CA). Carbapenem resistance in *P. aeruginosa* is commonly mediated by multifactorial non-carbapenemase mechanisms against which the ATM–CZA combination is not always effective. It has been shown to be effective in select scenarios, including for MDR infections caused by GES-producing *P. aeruginosa* (25). However, there are no molecular or phenotypic assays that can detect GES enzymes given their relatively low prevalence in the United States. Our study demonstrates that *P. aeruginosa* may yield major errors by the SX method. Thus, laboratories should be cautious when considering ATM–CZA susceptibility testing for *P. aeruginosa*.

**TABLE 6:** Recommendations for clinical laboratories to test susceptibility of the ATM–CZA combination

Organism	ATM–CZA susceptibility testing recommended (Y/N)	Isolates to test
Enterobacterales	Yes	Isolates producing or harboring genes encoding for metallo-β-lactamase enzymes Isolates confirmed to be non-susceptible to both ATM and CZA by phenotypic susceptibility testing
*P. aeruginosa*	No	-^*a*^
*S. maltophilia*	Yes	All isolates

^*a*^ -, no values set.

The limitations of our study include the lack of comparison between ETEST and MIC test strips (Liofilchem, Italy), which previously demonstrated potential to be accurate and reliable with the SX method (22). A larger multicenter study is warranted for further thorough assessment of the performance of the SX method.

Due to reports of emerging resistance in MBL-producing Enterobacterales, susceptibility testing is warranted for ATM–CZA when the combination is being considered for treatment and for ATM–avibactam when the single drug is available (18, 19, 21). Thus, *in vitro* testing can guide the rational use of the combination for treatment (Table 6). Our study demonstrates that the SX method is accurate, precise, and feasible for clinical laboratories to perform ATM–CZA susceptibility testing for multidrug-resistant gram-negative pathogens.

## References

[1] Doi Y. 2019. Treatment options for carbapenem-resistant gram-negative bacterial infections. Clinical Infectious Diseases 69:S565–S575. doi:10.1093/cid/ciz830 31724043 PMC6853760

[2] Alraddadi BM, Saeedi M, Qutub M, Alshukairi A, Hassanien A, Wali G. 2019. Efficacy of ceftazidime-avibactam in the treatment of infections due to carbapenem-resistant enterobacteriaceae. BMC Infect Dis 19:772. doi:10.1186/s12879-019-4409-1 31484510 PMC6724371

[3] Torres A, Wible M, Tawadrous M, Irani P, Stone GG, Quintana A, Debabov D, Burroughs MW, Bradford PA, Kollef M. 2023. Efficacy and safety of ceftazidime/avibactam in patients with infections caused by β-lactamase-producing gram-negative pathogens: a pooled analysis from the phase 3 clinical trial programme. J Antimicrob Chemother 78:2672–2682. doi:10.1093/jac/dkad280 37700689 PMC11157139

[4] Motsch J, Köksal I, Murta de Oliveira C, Kartsonis NA, Butterton JR, Paschke A, Young K K, Kaye KS. 2020. RESTORE-IMI 1: a multicenter, randomized, double-blind trial comparing efficacy and safety of imipenem/relebactam vs colistin plus Imipenem in patients with imipenem-nonsusceptible bacterial infections. Clinical Infectious Diseases 70:1799–1808. doi: 10.1093/cid/ciz530 31400759 10.1093/cid/ciz530 PMC7156774

[5] Kaye KS, Bhowmick T, Metallidis S, Bleasdale SC, Sagan OS, Stus V, Vazquez J, Zaitsev V, Bidair M, Chorvat E, Dragoescu PO, Fedosiuk E, Horcajada JP, Murta C, Sarychev Y, Stoev V, Morgan E, Fusaro K, Griffith D, Lomovskaya O, Alexander EL, Loutit J, Dudley MN, Giamarellos-Bourboulis EJ. 2018. Effect of meropenem-vaborbactam vs piperacillin-tazobactam on clinical cure or improvement and microbial eradication in complicated urinary tract infection: the TANGO I randomized clinical trial. JAMA 319:788–799. doi:10.1001/jama.2018.0438 29486041 PMC5838656

[6] van Duin D, Lok JJ, Earley M, Cober E, Richter SS, Perez F, Salata RA, Kalayjian RC, Watkins RR, Doi Y, Kaye KS, Fowler VG Jr, Paterson DL, Bonomo RA, Evans S. 2018. Colistin versus ceftazidime-avibactam in the treatment of infections due to carbapenem-resistant enterobacteriaceae. Clinical Infectious Diseases 66:163–171. doi:10.1093/cid/cix783 29020404 PMC5850032

[7] Wunderink RG, Giamarellos-Bourboulis EJ, Rahav G, Mathers AJ, Bassetti M, Vazquez J, Cornely OA, Solomkin J, Bhowmick T, Bishara J, Daikos GL, Felton T, Furst MJL, Kwak EJ, Menichetti F, Oren I, Alexander EL, Griffith D, Lomovskaya O, Loutit J, Zhang S, Dudley MN, Kaye KS. 2018. Effect and safety of meropenem-vaborbactam versus best-available therapy in patients with carbapenem-resistant enterobacteriaceae infections: the TANGO II randomized clinical trial. Infect Dis Ther 7:439–455. doi:10.1007/s40121-018-0214-1 30270406 PMC6249182

[8] Alosaimy S, Jorgensen SCJ, Lagnf AM, Melvin S, Mynatt RP, Carlson TJ, Garey KW, Allen D, Venugopalan V, Veve M, Athans V, Saw S, Yost CN, Davis SL, Rybak MJ. 2020 a. Real-world multicenter analysis of clinical outcomes and safety of meropenem-vaborbactam in patients treated for serious gram-negative bacterial infections. Open Forum Infect Dis 7:ofaa051. doi:10.1093/ofid/ofaa051 32161775 PMC7060146

[9] Alosaimy S, Jorgensen SCJ, Lagnf AM, Melvin S, Mynatt RP, Carlson TJ, Garey KW, Allen D, Venugopalan V, Veve M, Athans V, Saw S, Yost CN, Davis SL, Rybak MJ. 2020 b. Real-world multicenter analysis of clinical outcomes and safety of meropenem-vaborbactam in patients treated for serious gram-negative bacterial infections. Open Forum Infect Dis 7:faa051. doi:10.1093/ofid/ofaa051 PMC7060146 32161775

[10] Sader HS, Mendes RE, Carvalhaes CG, Kimbrough JH, Castanheira M. 2023. changing epidemiology of carbapenemases among carbapenem-resistant enterobacterales from united states hospitals and the activity of aztreonam-avibactam against contemporary enterobacterales (2019-2021). Open Forum Infect Dis 10:ofad046. doi:10.1093/ofid/ofad046 36846612 PMC9945928

[11] Reyes J, Komarow L, Chen L, Ge L, Hanson BM, Cober E, Herc E, Alenazi T, Kaye KS, Garcia-Diaz J, et al.. 2023. Global epidemiology and clinical outcomes of carbapenem-resistant Pseudomonas aeruginosa and associated carbapenemases (POP): a prospective cohort study. Lancet Microbe 4:e159–e170. doi:10.1016/S2666-5247(22)00329-9 36774938 PMC10016089

[12] Falcone M, Tiseo G, Carbonara S, Marino A, Di Caprio G, Carretta A, Mularoni A, Mariani MF, Maraolo AE, Scotto R, et al.. 2023. Mortality attributable to bloodstream infections caused by different carbapenem-resistant gram-negative bacilli: results from a nationwide study in italy (ALARICO Network). Clin Infect Dis 76:2059–2069. doi:10.1093/cid/ciad100 36801828

[13] Cai B, Tillotson G, Benjumea D, Callahan P, Echols R. 2020. The burden of bloodstream infections due to Stenotrophomonas maltophilia in the united states: a large, retrospective database study. Open Forum Infect Dis 7:ofaa141. doi:10.1093/ofid/ofaa141 32462047 PMC7240339

[14] Tamma PD, Aitken SL, Bonomo RA, Mathers AJ, van Duin D, Clancy CJ. 2023. Infectious diseases society of america 2023 guidance on the treatment of antimicrobial resistant gram-regative infections. Clinical Infectious Diseases ciad428. doi:10.1093/cid/ciad428 37463564

[15] Falcone M, Daikos GL, Tiseo G, Bassoulis D, Giordano C, Galfo V, Leonildi A, Tagliaferri E, Barnini S, Sani S, Farcomeni A, Ghiadoni L, Menichetti F. 2021. Efficacy of ceftazidime-avibactam plus aztreonam in patients with bloodstream infections caused by metallo-β-lactamase-producing enterobacterales. Clinical Infectious Diseases 72:1871–1878. doi:10.1093/cid/ciaa586 32427286

[16] Matsumoto T, Yuasa A, Miller R, Pritchard C, Ohashi T, Taie A, Gordon J. 2023. Estimating the economic and clinical value of introducing ceftazidime/avibactam into antimicrobial practice in Japan: a dynamic modelling study. Pharmacoecon Open 7:65–76. doi:10.1007/s41669-022-00368-w 36107306 PMC9476387

[17] The Health Insurance Review and Assessment Service (President Kang Jung-Gu). 2024. Publication of review result of the tenth pharmaceutical reimbursement evaluation committee Of2023. Korea

[18] Sadek M, Juhas M, Poirel L, Nordmann P. 2020. Genetic features leading to reduced susceptibility to aztreonam-avibactam among metallo-β-lactamase-producing Escherichia coli isolates. Antimicrob Agents Chemother 64:01659–20. doi:10.1128/AAC.01659-20 PMC7674043 32988825

[19] Sader HS, Castanheira M, Kimbrough JH, Kantro V, Mendes RE. 2023. Aztreonam/avibactam activity against a large collection of carbapenem-resistant enterobacterales (CRE) collected in hospitals from Europe. Asia and Latin America JAC Antimicrob Resist 5:dlad032. 10.1093/jacamr/dlad032 PMC10032302 36968952

[20] Wu S, Zong Z. 2022. Aztreonam-avibactam: an option against carbapenem-resistant enterobacterales with emerging resistance. Precis Clin Med 5:pbac029. doi:10.1093/pcmedi/pbac029 PMC9745765 36519138

[21] Mendes RE, Doyle TB, Streit JM, Arhin FF, Sader HS, Castanheira M. 2021. Investigation of mechanisms responsible for decreased susceptibility of aztreonam/avibactam activity in clinical isolates of Enterobacterales collected in Europe, Asia and Latin America in 2019.Journal of Antimicrobial Chemotherapy 76:2833–2838. doi:10.1093/jac/dkab279 34436603 PMC8561256

[22] Khan A, Erickson SG, Pettaway C, Arias CA, Miller WR, Bhatti MM. 2021. Evaluation of susceptibility testing methods for aztreonam and ceftazidime-avibactam combination therapy on extensively drug-resistant gram-negative organisms. Antimicrob Agents Chemother 65:00846–21. doi:10.1128/AAC.00846-21 PMC8522751 34424044

[23] Harris H, Tao L, Jacobs EB, Bergman Y, Adebayo A, Tekle T, Lewis S, Dahlquist A, Abbey TC, Wenzler E, Humphries R, Simner PJ. 2023. Multicenter evaluation of an MIC-based aztreonam and ceftazidime-avibactam broth disk elution test. J Clin Microbiol 61:e01647–22. doi:10.1128/jcm.01647-22 37070979 PMC10204635

[24] Clinical Laboratory and Standards Institute. 2023. Development of In Vitro Susceptibility Test Methods, Breakpoints, and Quality Control Parameters. 6th ed

[25] Khan A, Tran TT, Rios R, Hanson B, Shropshire WC, Sun Z, Diaz L, Dinh AQ, Wanger A, Ostrosky-Zeichner L, Palzkill T, Arias CA, Miller WR. 2019. Extensively drug-resistant Pseudomonas aeruginosa ST309 harboring tandem guiana extended spectrum β-lactamase enzymes: a newly emerging threat in the united states. Open Forum Infect Dis 6:ofz273. doi:10.1093/ofid/ofz273 31281867 PMC6602888

[26] Clinical laboratory and standards institute. 2024. CLSI M07 Methods for Dilution Antimicrobial Susceptibility Tests for Bacteria That Grow Aerobically. 12th ed

[27] A KJ M KK, M de JBL M c F SD SD, A HM. 2017. In Vitro activity of aztreonam-avibactam against enterobacteriaceae and Pseudomonas aeruginosa isolated by clinical laboratories in 40 countries from 2012 to 2015. Antimicrob Agents Chemother 61:10.1128/aac.00472-17. doi: 10.1128/AAC.00472-17 10.1128/AAC.00472-17 PMC5571336 28630192

[28] Sader HS, Duncan LR, Arends SJR, Carvalhaes CG, Castanheira M. 2020. Antimicrobial activity of aztreonam-avibactam and comparator agents when tested against a large collection of contemporary stenotrophomonas maltophilia isolates from medical centers worldwide. Antimicrob Agents Chemother 64:e01433-20. doi:10.1128/AAC.01433-20 32900683 PMC7577171

[29] Clinical laboratory and standards institute. 2024. Performance standards for 520 antimicrobial susceptibility testing. 34th ed

